# Supplementary material for: Comparative Analysis of miRNA Abundance Revealed the Function of Vvi-miR828 in Fruit Coloring in Root Restriction Cultivation Grapevine (Vitis vinifera L.)
Source: Int J Mol Sci. 2019 Aug 20;20(16):4058. doi: 10.3390/ijms20164058 (PMC6720769; doi:10.3390/ijms20164058)
Supplement: Supplementary file 1 [file ijms-20-04058-s001.zip › Supplementary files legents.docx]

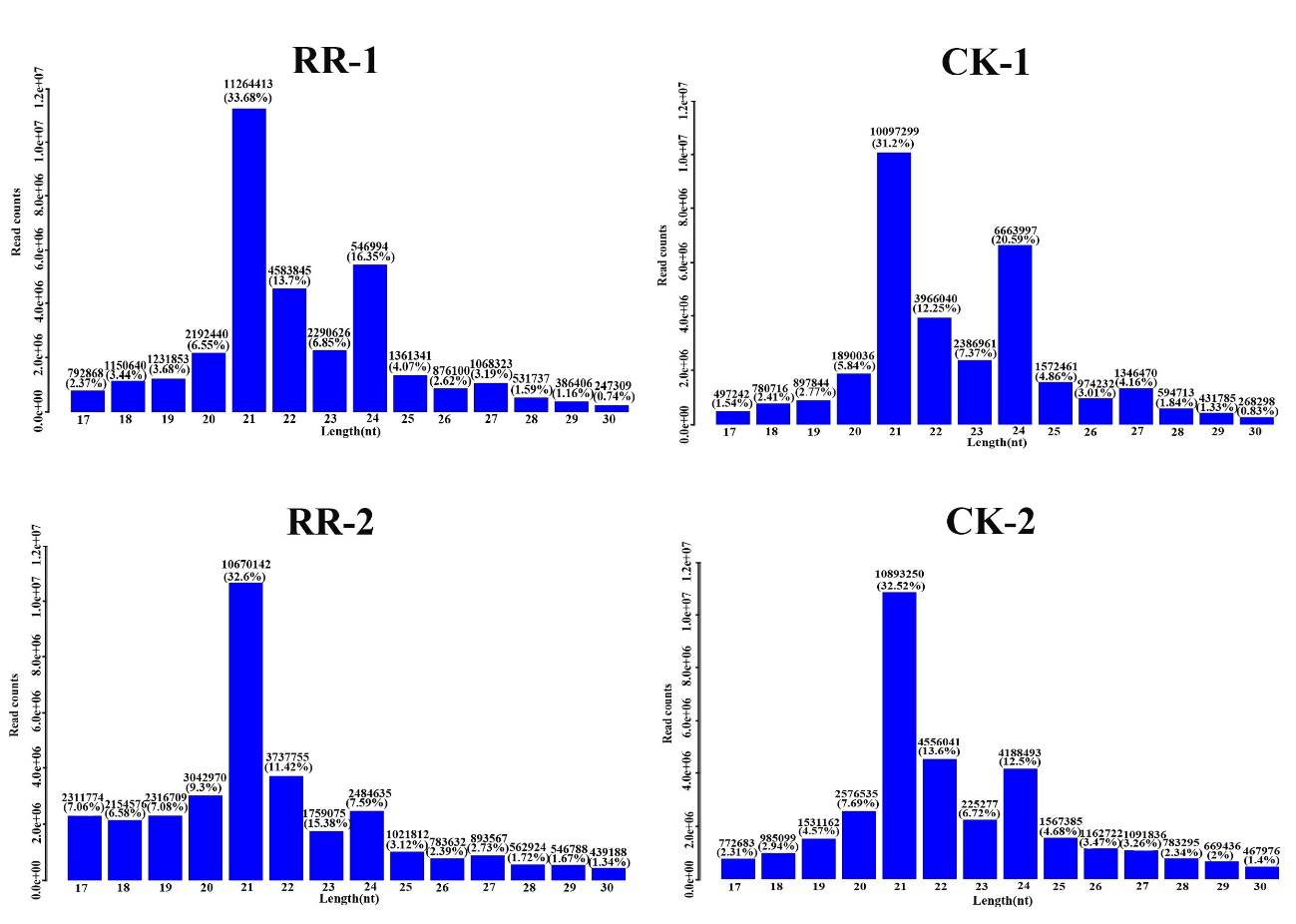


**Figure S1.** Reads copy number and length distribution.


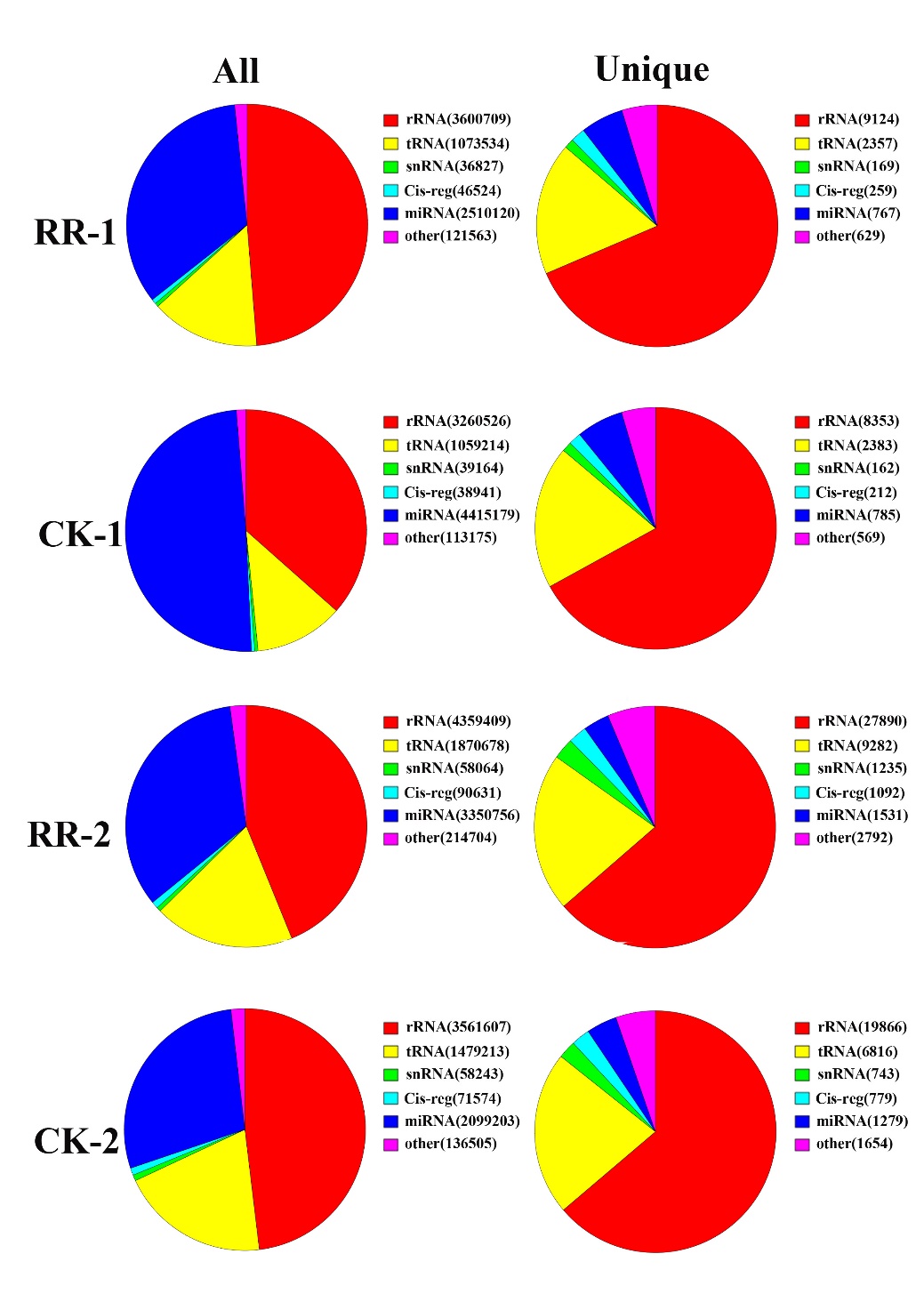


**Figure S2.** Distribution of sRNA in each samples.


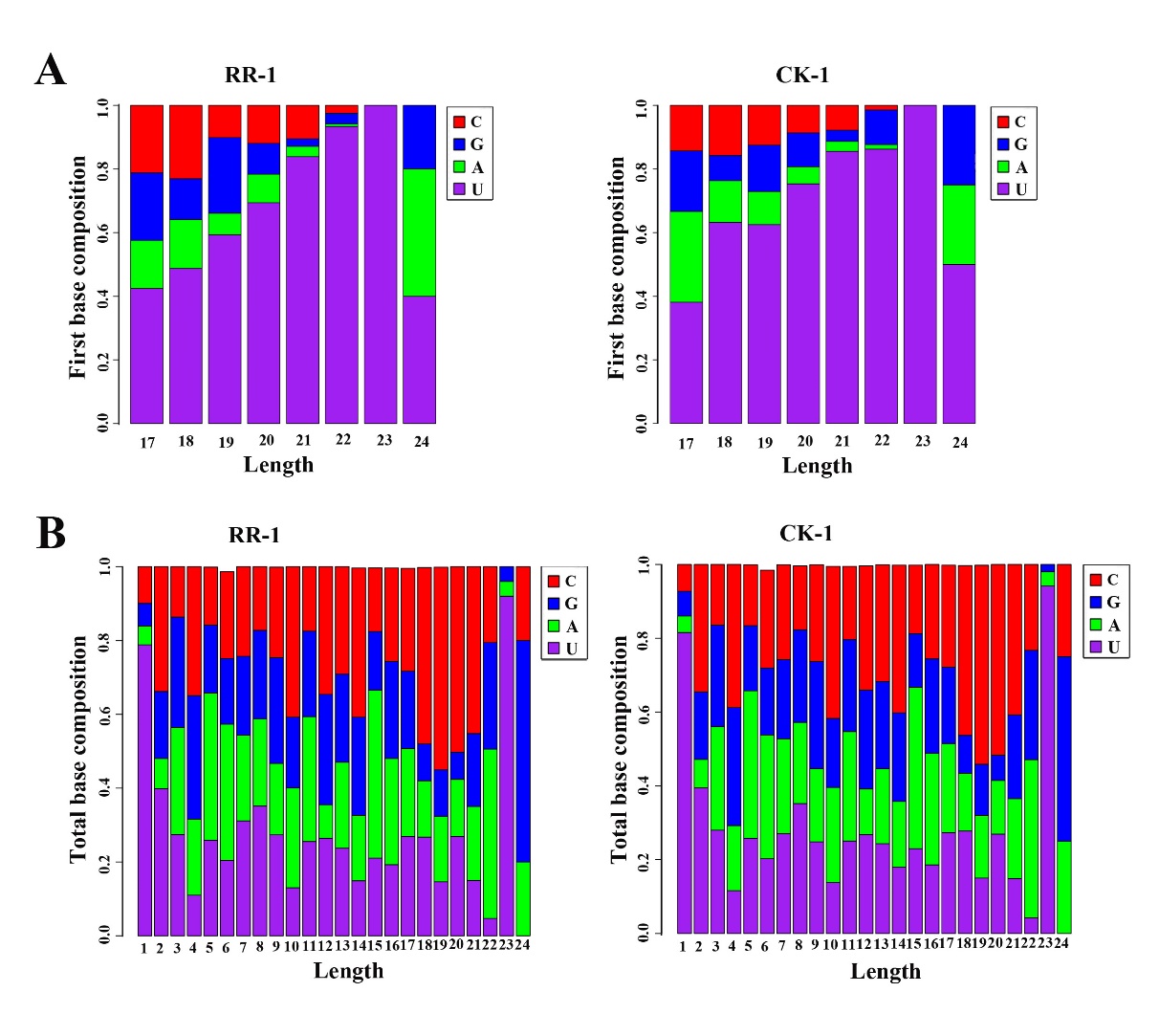


**Figure S3. miRNA variants and their nucleotide bias position in RR-1 and CK-1 libraries.** (A) First nucleotide bias for the first position of 17 to 24 nt mRNAs. Nucleotide U predominates. (B) miRNA nucleotide bias for each position of 24 nt miRNAs.


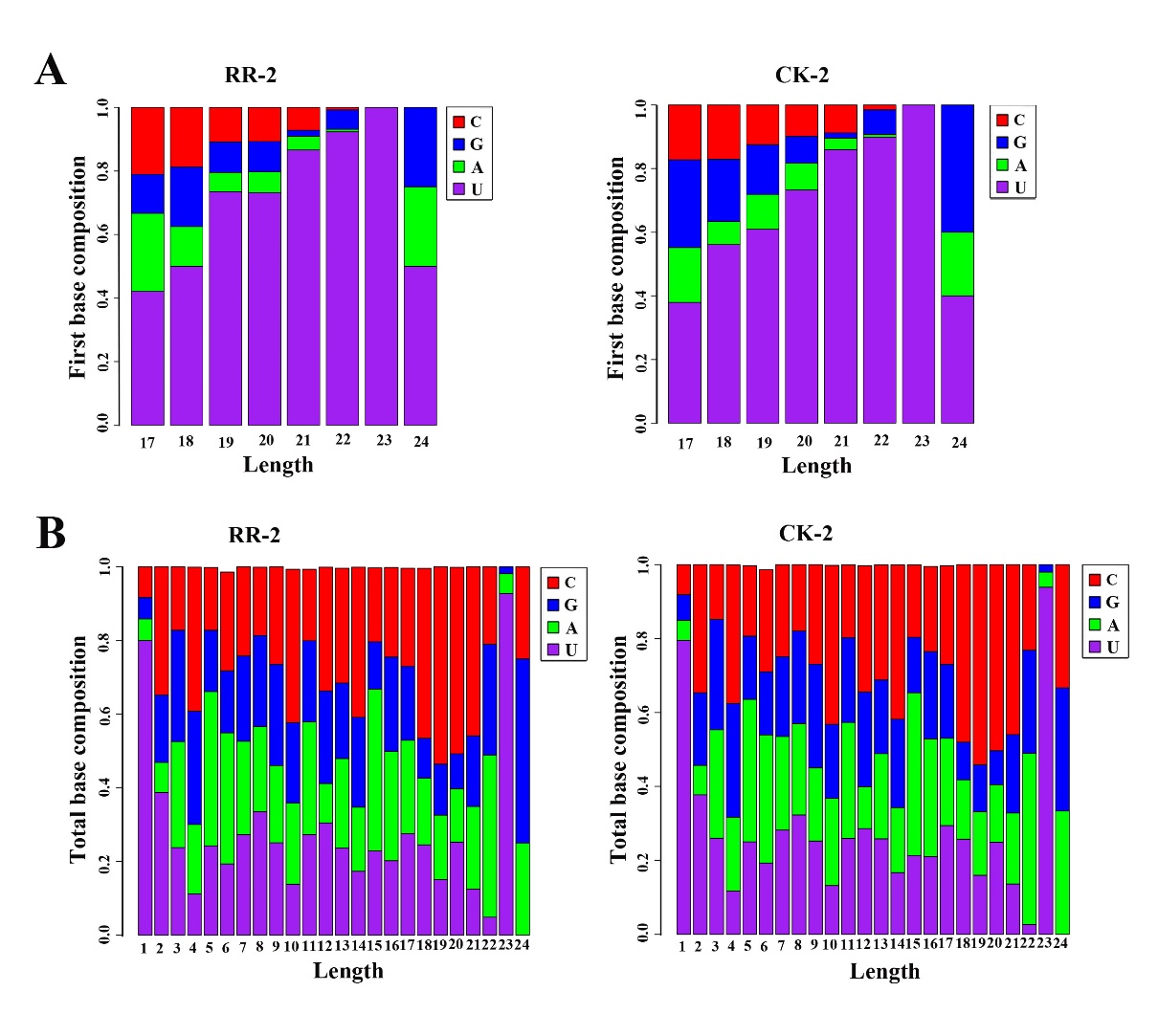


**Figure S4. miRNA variants and their nucleotide bias position in RR-2 and CK-2 libraries.** (A) First ucleotide bias for the first position of 17 to 24 nt mRNAs. Nucleotide U predominates. (B) miRNA nucleotide bias for each position of 24 nt miRNAs.


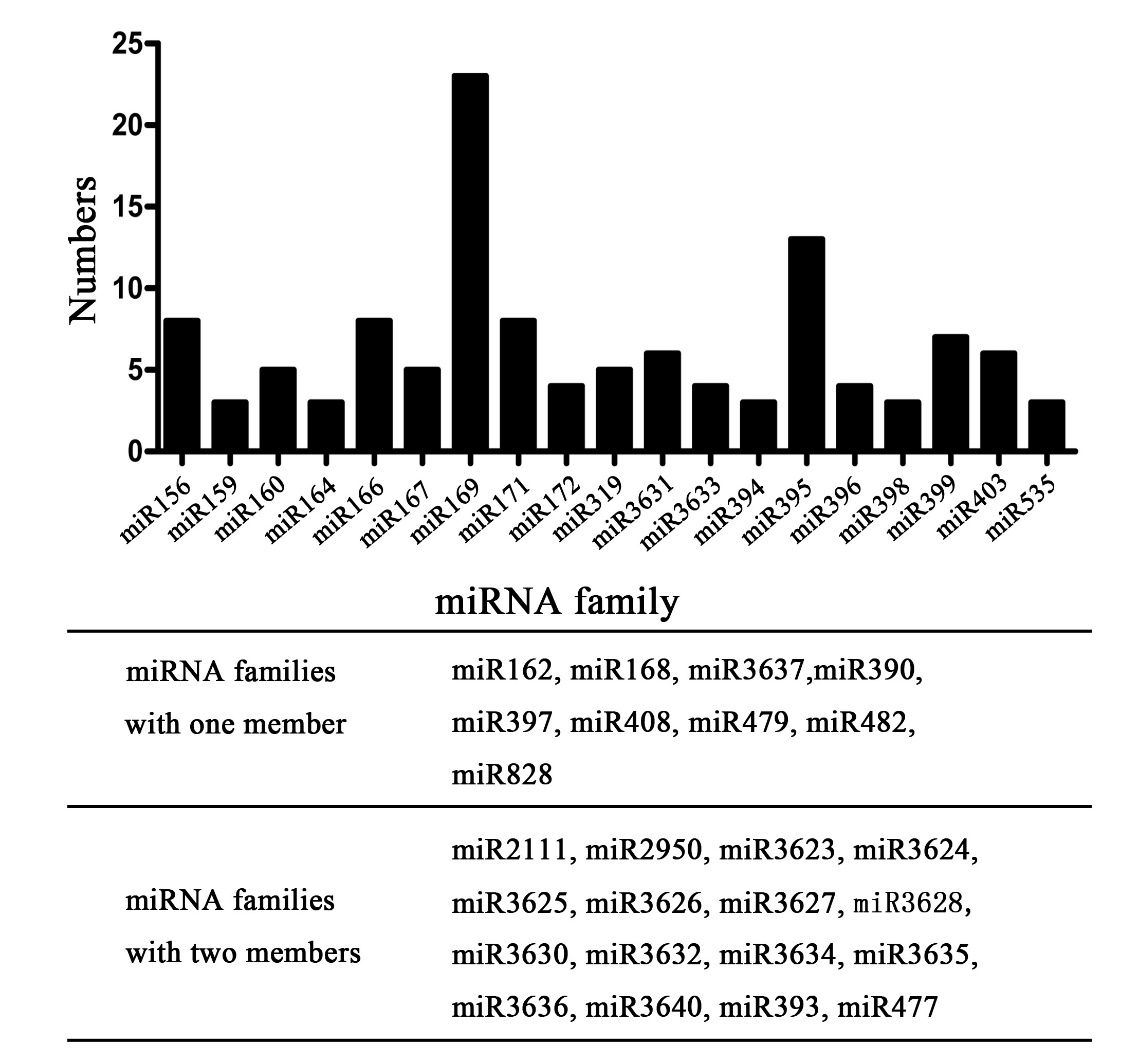


**Figure S5.** Count of miRNAs in different miRNA families.


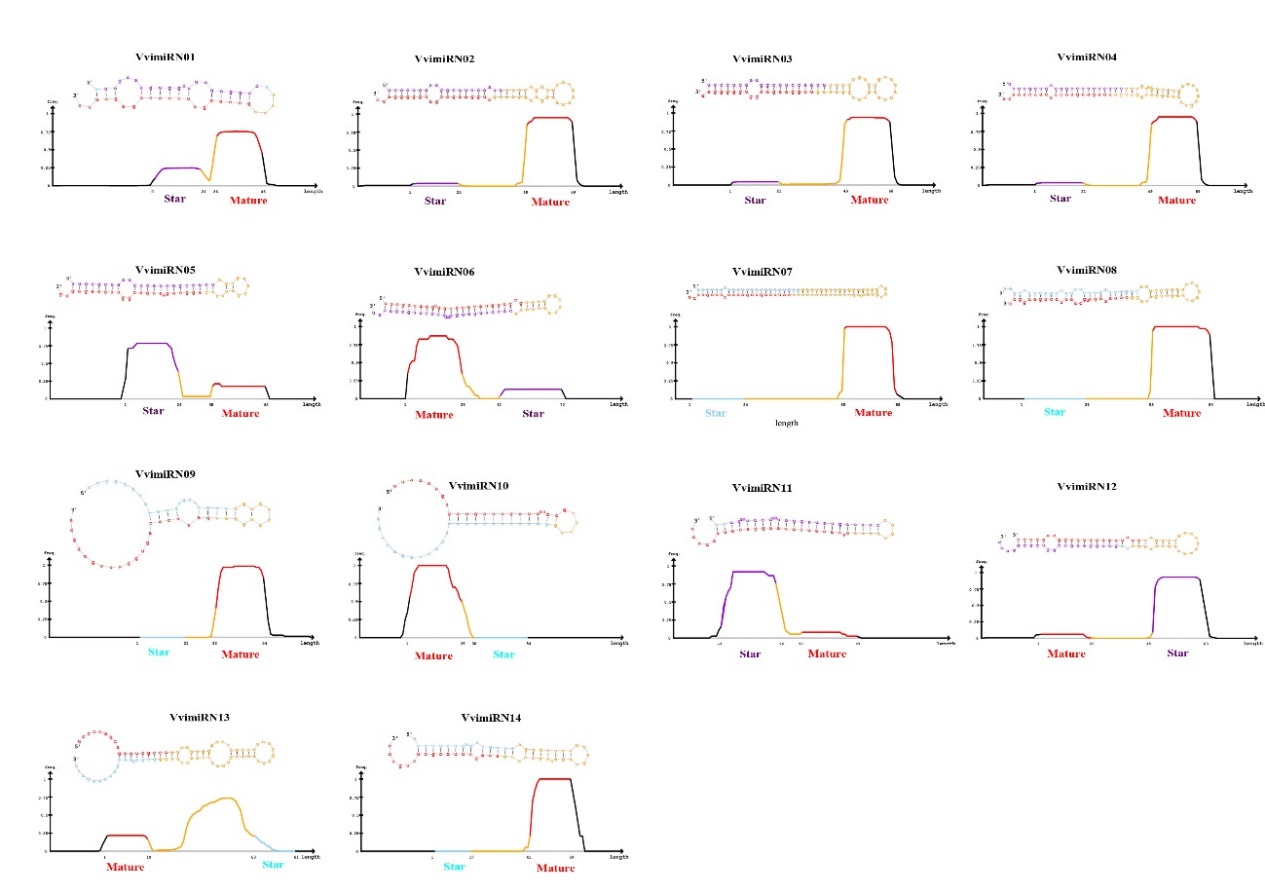


**Figure S6.** Predicted precursor structures of novel miRNAs found in grape fruits.


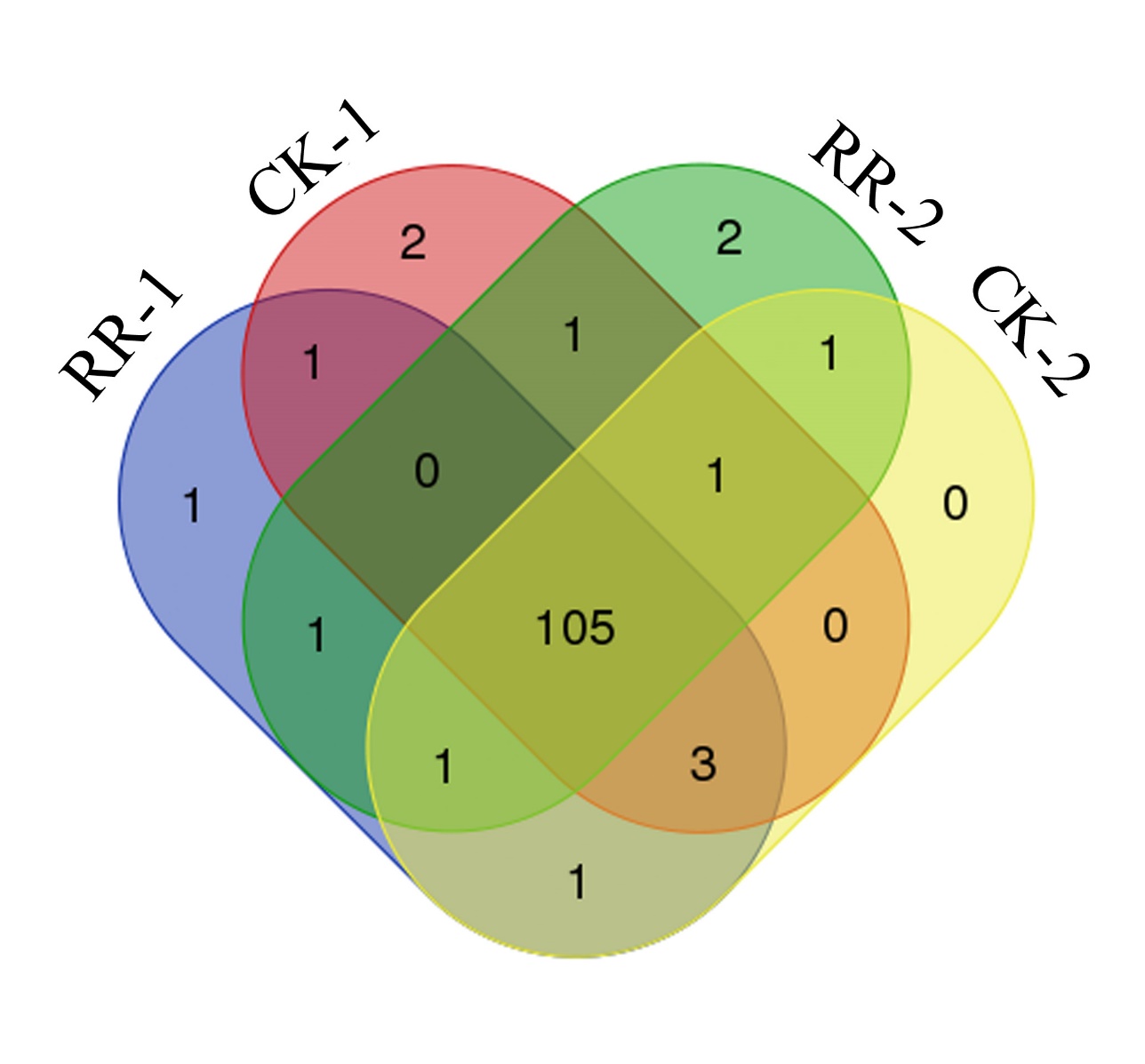


**Figure S7.** Venn diagram of miRNAs expressed in different samples.

**Table S1 Categorization of reads of small RNAs in grape fruit at two developmental stages in two groups.**

**Table S2 Analysis of small RNAs from grape fruits of 'Muscat ' under restriction root and control group.**

**Table S3 Numbers and percentage of reads for each small RNA classification identified in four libraries.**

**Table S4 Detailed information of identified novel miRNAs.**

**Table S5 The expression of conserved miRNAs in four libraries.**

**Table S6 The expression of novel miRNAs.**

**Table S7 The predicted target genes of conserved miRNAs.**

**Table S8 The predicted target genes of novel miRNAs.**

**Table S9 The prime lists used in this study.**
