# Supplementary figures and images for: Comparative Analysis of miRNA Abundance Revealed the Function of Vvi-miR828 in Fruit Coloring in Root Restriction Cultivation Grapevine (Vitis vinifera L.)
Source: Int J Mol Sci. 2019 Aug 20;20(16):4058. doi: 10.3390/ijms20164058 (PMC6720769; doi:10.3390/ijms20164058)

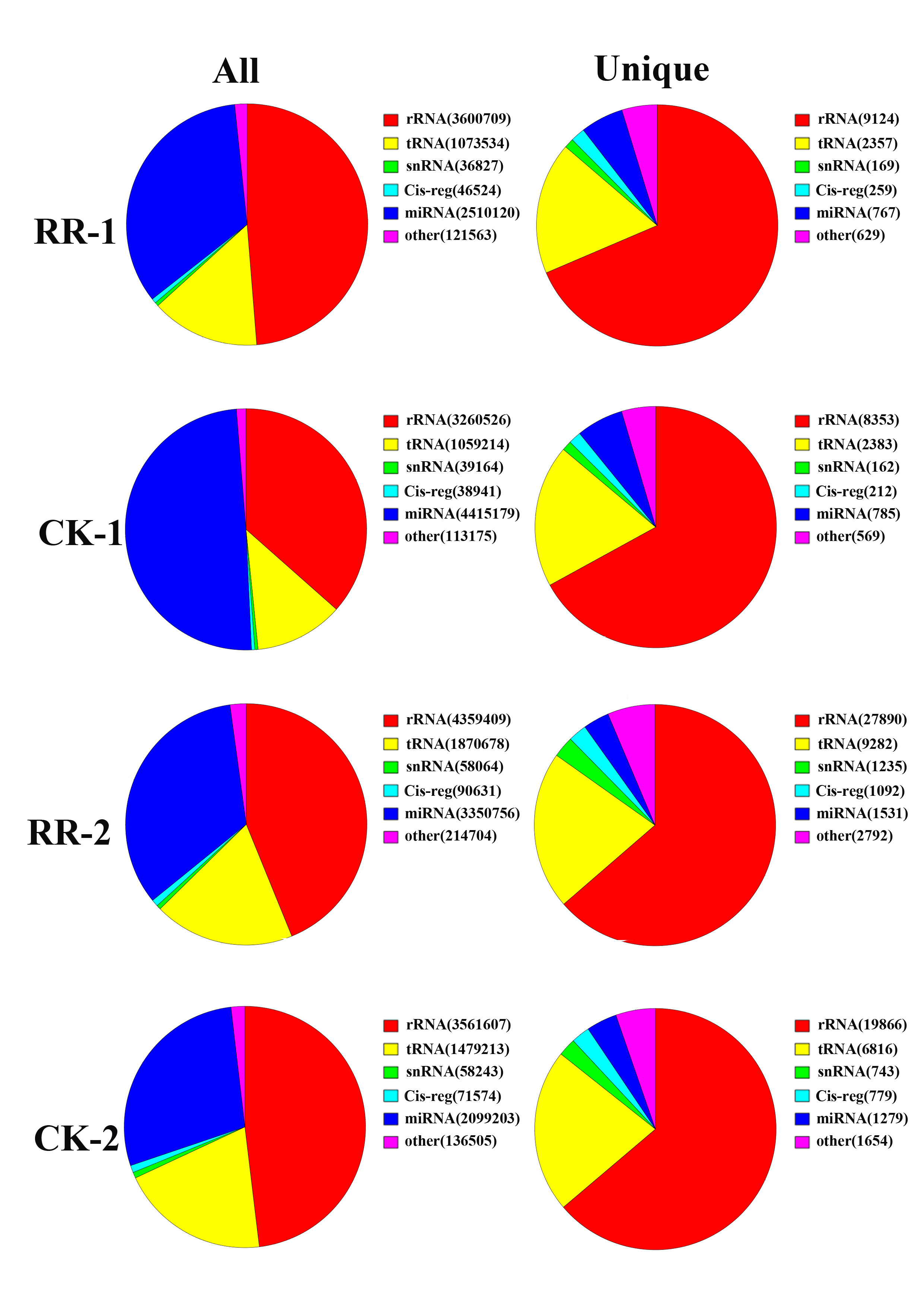

Supplement: Supplementary file 1 [file ijms-20-04058-s001.zip › Fig.S2.jpg]

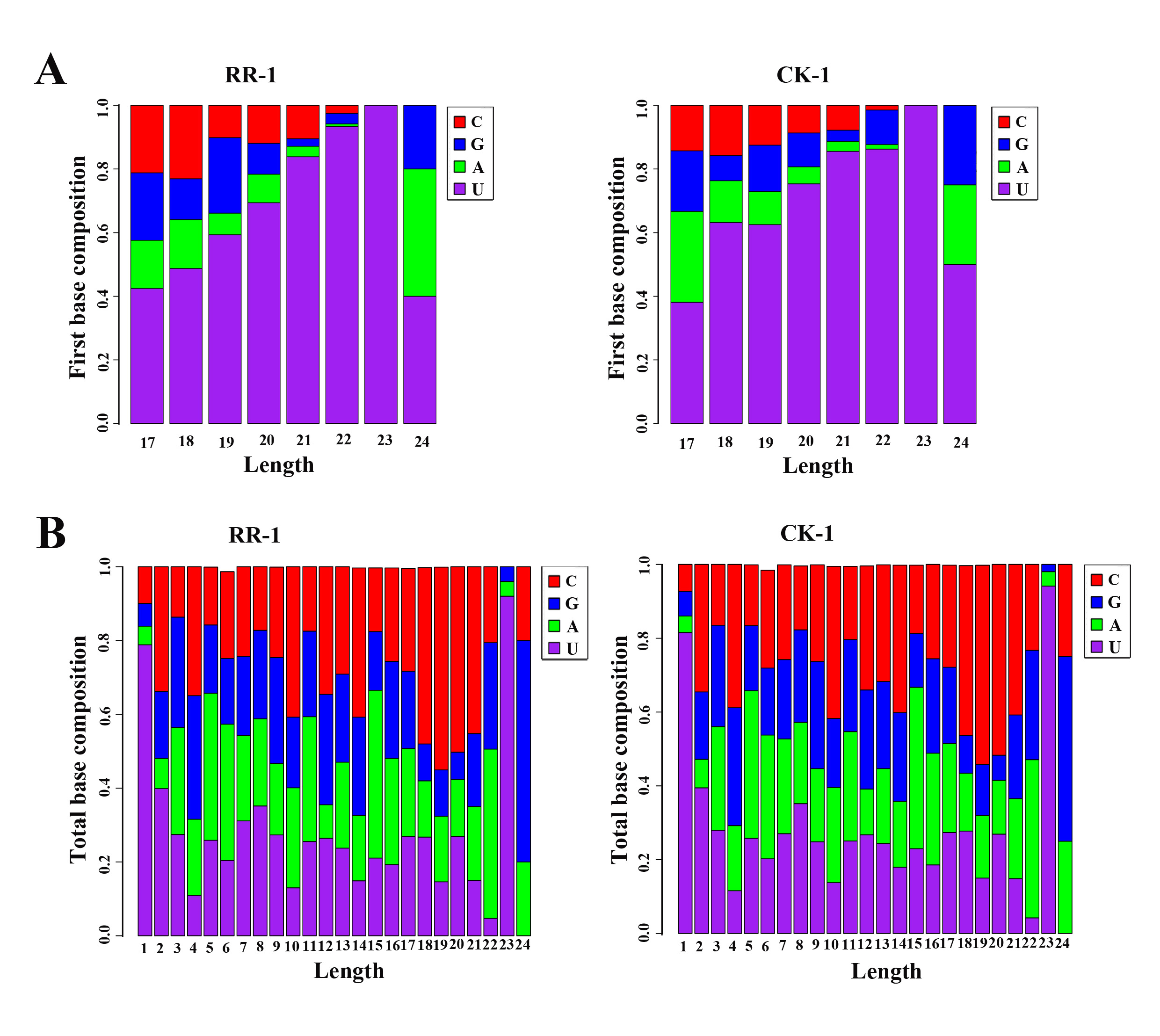

Supplement: Supplementary file 1 [file ijms-20-04058-s001.zip › Fig.S3.jpg]

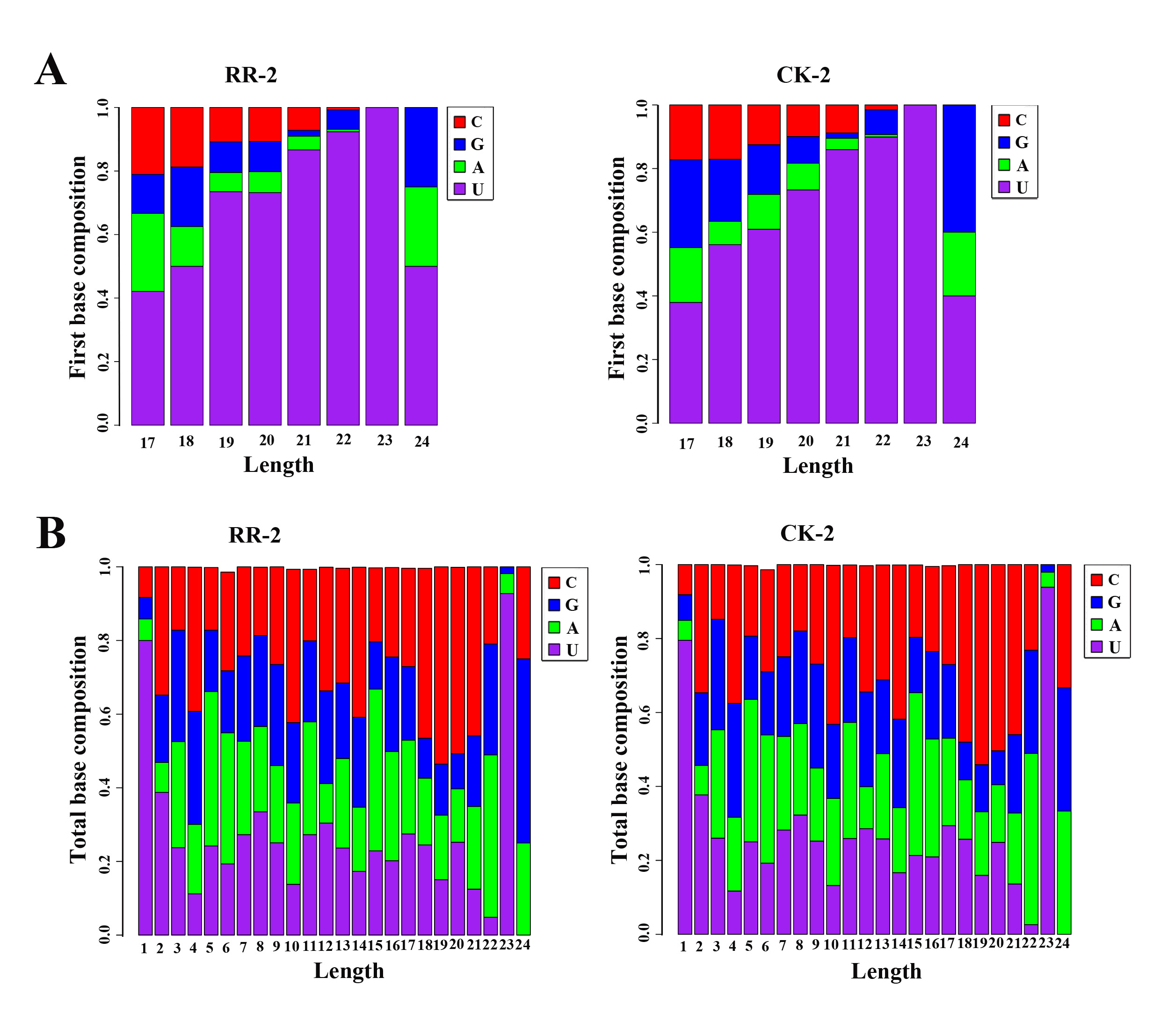

Supplement: Supplementary file 1 [file ijms-20-04058-s001.zip › Fig.S4.jpg]

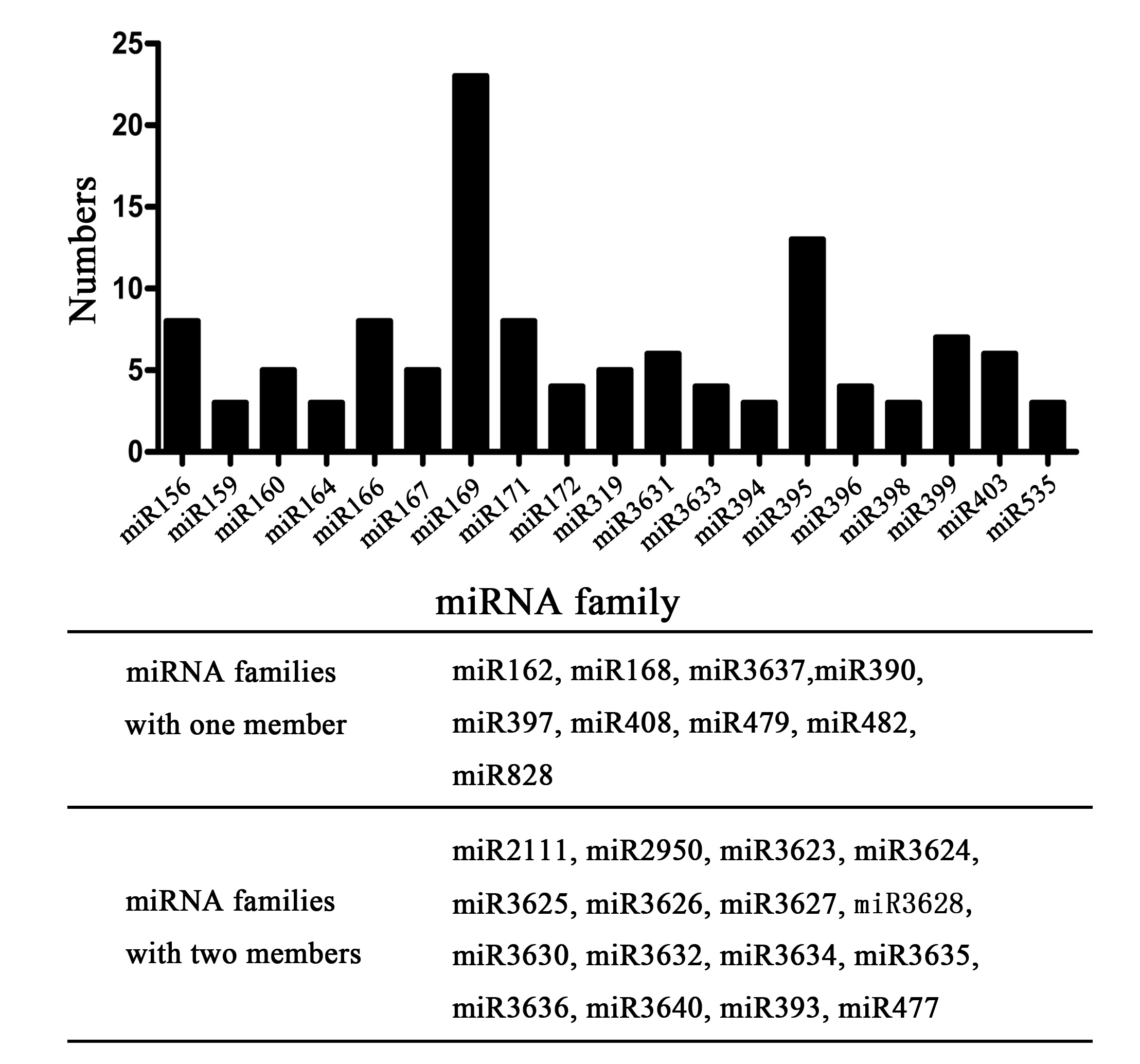

Supplement: Supplementary file 1 [file ijms-20-04058-s001.zip › Fig.S5.jpg]

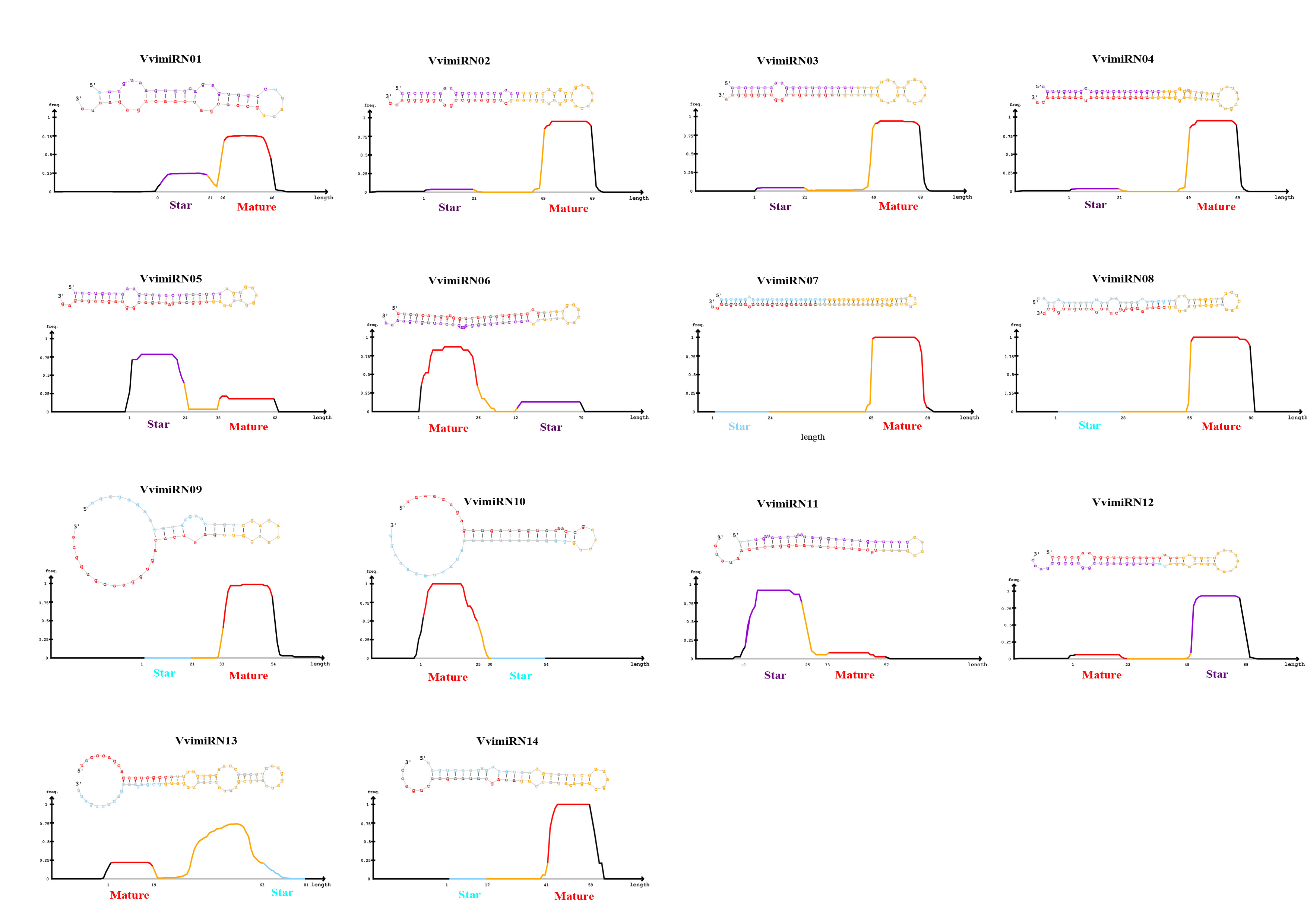

Supplement: Supplementary file 1 [file ijms-20-04058-s001.zip › Fig.S6.jpg]

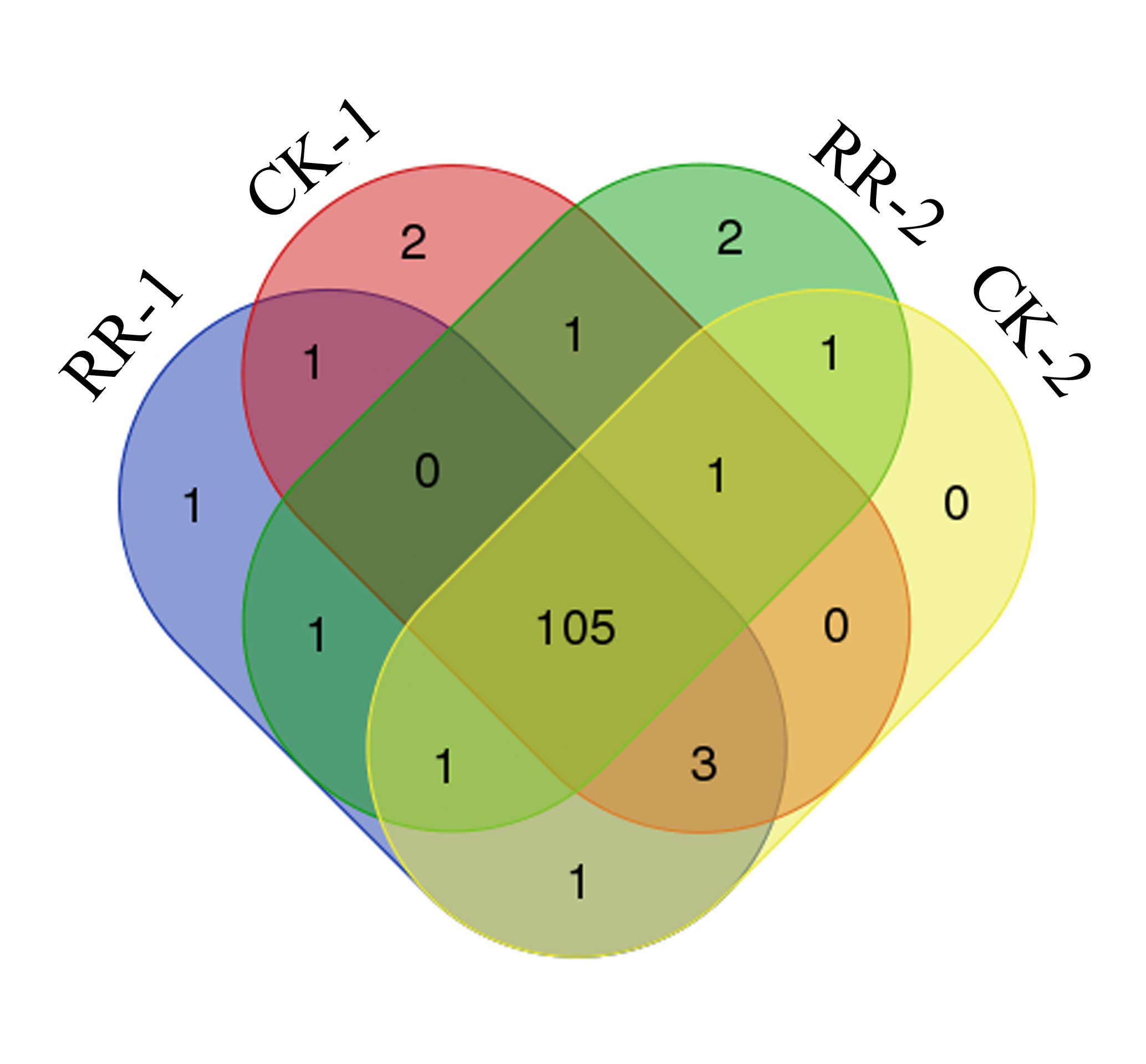

Supplement: Supplementary file 1 [file ijms-20-04058-s001.zip › Fig.S7.jpg]

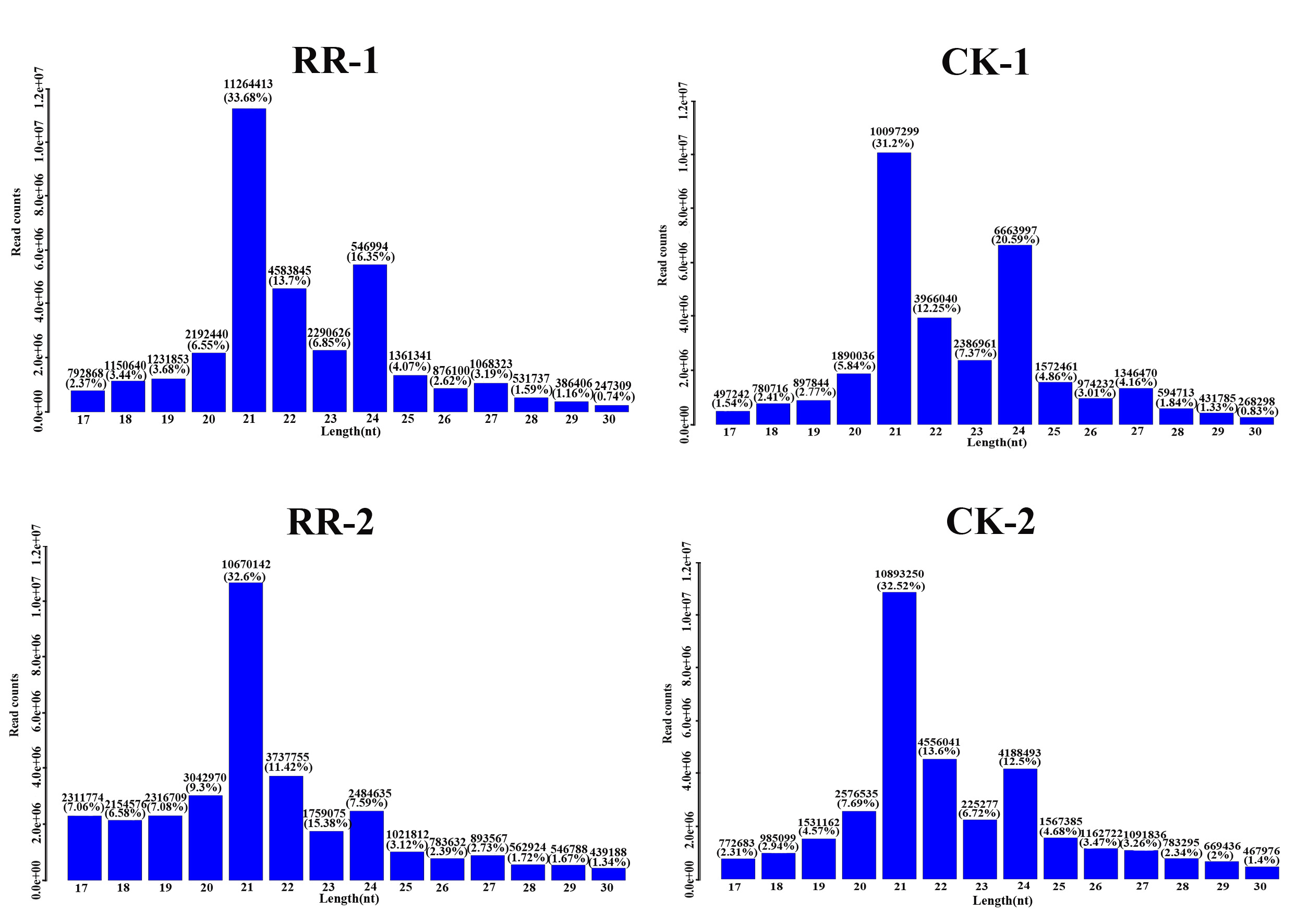

Supplement: Supplementary file 1 [file ijms-20-04058-s001.zip › Fig.S1.jpg]
